# Dynamic Functional Connectivity Strength Within Different Frequency-Band in Schizophrenia

**DOI:** 10.3389/fpsyt.2019.00995

**Published:** 2020-02-12

**Authors:** Yuling Luo, Hui He, Mingjun Duan, Huan Huang, Zhangfeng Hu, Hongming Wang, Gang Yao, Dezhong Yao, Jianfu Li, Cheng Luo

**Affiliations:** The Clinical Hospital of Chengdu Brain Science Institute, MOE Key Lab for Neuroinformation, High-Field Magnetic Resonance Brain Imaging Key Laboratory of Sichuan Province, University of Electronic Science and Technology of China, Chengdu, China

**Keywords:** Schizophrenia, dynamic functional connectivity strength, rest-state functional magnetic resonance imaging, different, frequency band

## Abstract

As a complex psychiatric disorder, schizophrenia is interpreted as a “dysconnection” syndrome, which is linked to abnormal integrations in between distal brain regions. Recently, neuroimaging has been widely adopted to investigate how schizophrenia affects brain networks. Furthermore, some studies reported frequency dependence of the abnormalities of functional network in schizophrenia, however, dynamic functional connectivity with frequency dependence is rarely used to explore changes in the whole brain of patients with schizophrenia (SZ). Therefore, in the current study, dynamic functional connectivity strength (dFCS) was performed on resting-state functional magnetic resonance data from 96 SZ patients and 121 healthy controls (HCs) at slow-5 (0.01–0.027 Hz), slow-4 (0.027–0.073 Hz), slow-3 (0.073–0.198 Hz), and slow-2 (0.198–0.25 Hz) frequency bands and further assessed whether the altered dFCS was correlated to clinical symptoms in SZ patients. Results revealed that decreased dFCS of schizophrenia were found in salience, auditory, sensorimotor, visual networks, while increased dFCS in cerebellum, basal ganglia, and prefrontal networks were observed across different frequency bands. Specifically, the thalamus subregion of schizophrenic patients exhibited enhanced dynamic FCS in slow-5 and slow-4, while reduced in slow-3. Moreover, in slow-5 and slow-4, significant interaction effects between frequency and group were observed in the left calcarine cortex, the bilateral inferior orbitofrontal gyrus, and anterior cingulum cortex (ACC). Furthermore, the altered dFCS of insula, thalamus (THA), calcarine cortex, orbitofrontal gyrus, and paracentral lobule were partial correlated with clinical symptoms of SZ patients in slow-5 and slow-4 bands. These results demonstrate the abnormalities of dFCS in schizophrenia patients is rely on different frequency bands and may provide potential implications for exploring the neuropathological mechanism of schizophrenia.

## Introduction

Schizophrenia (SZ) is a chronic and diverse pathological psychiatric disorder. It typically emerges during adolescence or young adulthood (15–35 years old), and clinically characterized by emotional, attentional, and cognitive dysfunction, accompanied by some symptoms such as hallucinations and delusions. Yet, neural mechanism and pathogenesis of SZ remain poorly understood. Converging studies suggest that the pathology of SZ is related to abnormal structure and functional of large- scale brain network (1–3).

The development and application of functional magnetic resonance imaging (fMRI) has shedded a new light on exploring the pathogenesis of SZ. Findings from fMRI studies have reported abnormal low-frequency (0.01–0.08 Hz) functional connectivity of SZ patients in the default mode network (DMN) (4), sensorimotor network (SMN) (5–7), salience network (SN) (8). Furthermore, based on earlier neurophysiological studies, Gohel and colleagues observed the resting-state functional connectivity of brain regions was differently across diverse frequency bands (9, 10). Moreover, previous studies had demonstrated that blood-oxygen-level-dependent imaging (BOLD) fMRI fluctuations >0.1 Hz had physiological significance (10, 11). While, most previous findings were observed within low frequency band functional networks (12). For example, Yu et al. (2014) researched frequency-specific alternation in SZ patients using the amplitude of low-frequency fluctuations (ALFF) (13). Zou et al. (2019) employed multi-frequency dynamic functional connectivity (dFC) to classify SZ patients from normal controls in low-frequency (14). Taken together, multiple frequency-dependent and higher-frequency brain network information would further provide the evidence to explore underlying etiology and mechanisms of SZ.

Prior neuroimaging researchers had found that distinct oscillator with specific properties and physiological functions could produce independent frequency bands. That was to say physiological signals of different frequency bands were generated by different functional regions of the brain (9). And physiological signals in the same brain network might be competing or cooperating with each other in different frequency bands (15). For instance, Zuo et al. (2010) found that the ALFF of basal ganglia network in slow-5 (0.01–0.027 Hz) were lower than that in slow-4 (0.027–0.073 Hz) frequency band (16).

Previous studies had focused on the analysis the static functional connectivity (sFC) of brain network in multiple-frequency of SZ. Recently, increasing evidence suggested that sFC was insufficient to explain the time-varying dynamic information interactions of brain (17–21), and dFC could offer much more nuanced characterization of dynamic brain function on a time scale (22, 23). The traditional and most widely used method for estimating dFC is the “sliding windows,” which divide the scan session into different sub-interval or windows (24–26). Using a sliding-window analysis, Sakoglu et al. (2010) evaluated dynamic changes of brain network in SZ when task stimulus (26). In recent years, a growing body of research employed dFC analysis to explore changes in neural activity patterns of patients with SZ. Dynamic multiple-frequency brain network analysis may further provide more evidence to explore pathogeny of SZ.

Based on evidence from the research of patients with SZ and functional neuroimaging studies, we hypothesized that the brain network connections were frequency dependence in SZ patients, and these abnormal dynamic frequency-specific might associated with sensory motor, visual processing, auditory, salience networks. To investigate this issue, the current study will measure the frequency-specific variability of large-scale functional network in SZ subjects. Studies had shown that functional connectivity strength (FCS) metric was closely related to physiological measures such as glucose metabolism, oxygen and regional cerebral blood flow (27, 28). Therefore, FCS metric can be used as an indicator to examine hub connectivity in brain network. In our study, FCS analysis was employed to investigate altered patterns of SZ patients compared to healthy controls (HCs) at four frequency bands (slow-5: 0.01–0.027 Hz, slow-4: 0.027–0.073 Hz, slow-3: 0.073–0.198 Hz, slow-2: 0.198–0.25 Hz) (16). Altered frequency-dependent dFCS might provide more insight into the neurobiological processes of schizophrenia.

## Materials and Methods

### Participants

Ninety-six patients with schizophrenia and 121 gender and age-matched healthy controls were obtained from our dataset, in which participants were recruited in the Clinical Hospital of Chengdu Brain Science Institute. All patients were diagnosed using the structured clinical interview for the DSM-IV axis I disorders-clinical version (SCID-I-CV), and they were taking the medication (e.g., antipsychotics). We excluded subjects with a history of brain damage, a history of substance-related illness, a major medical or neurological disease. The positive and negative symptom scale (PANSS) were employed to assess the severity of symptoms in patients with schizophrenia. All of data were included in our previous study (29), however, whole brain dFCS with frequency dependence were first evaluated in the dataset, differ from Dong's study. The Ethics Committee of the Clinical Hospital of Chengdu Brain Science Institute approved the study in accordance with the Helsinki Declaration. Written assents were obtained from all subjects.

### Data Acquisition

All subjects underwent structural and functional MRI scan on a 3-T scanner (GE DISCOVERY MR 750, USA) with 32 channel head coil at the University of Electronic Science and Technology of China. Soft foam and earplugs were used to fix subjects head and reduce scanning noise during the scanning process. High-resolution T1-weighted images were acquired by a three-dimensional fast spoiled gradient-echo (T1-3D FSPGR) sequence [repetition time (TR) = 6.008 ms, flip angle (FA) =9, matrix = 256 × 256, field of view (FOV) = 256 × 256 mm2, slice thickness = 1 mm, no gap, 152 slices]. Resting state functional images were performed with a gradient-echo echo planar sequence [TR =2,000 ms, echo time (TE) = 30 ms, FA =90, matrix = 64×64, FOV = 240×240 mm^2^, slice thickness = 4 mm, gap = 0.4 mm, number of slice = 35, scanning time = 510 s (255 volumes )]. All subjects were instructed to relax with eyes closed and think of nothing in particular, then kept their head not move as little as possible and without falling asleep.

### Data Preprocessing

Image preprocessing was conducted using NIT (http://www.neuro.uestc.edu.cn/NIT.html) software (30). Similar our previous research, normal data preprocessing steps were included in this study as following (31): 1) removing the first ten volumes; 2) slice timing; 3) realignment; 4) spatial normalization of the functional images was performed using 3D T1-based transformation. We coregistered individual 3D T1 images to functional images. The 3D T1 images were segmented and normalized to Montreal Neurologic Institute (MNI) space by a 12-parameter nonlinear transformation. 5) Filtering with a typical temporal bandpass, including slow-5 bandpass (0.001–0.027 Hz), slow-4 (0.027–0.073 Hz), slow-3 (0.073–0.198 Hz), and slow-2 (0.198–0.25 Hz) respectively. The global signal was not regressed out, as has been recently suggested in processing the schizophrenia functional data (32). In addition, we assessed framewise displacement (FD) of each subject using the following formula (33):

$$\text{FD}=\frac{1}{M-1}\sum_{i=2}^{M}\sqrt{{\left|\Delta t_{x_{i}}\right|}^{2}+{\left|\Delta t_{y_{i}}\right|}^{2}+{\left|\Delta t_{z_{i}}\right|}^{2}+{\left|\Delta d_{x_{i}}\right|}^{2}+{\left|\Delta d_{y_{i}}\right|}^{2}+{\left|\Delta d_{z_{i}}\right|}^{2}},$$

where *M* is the length of time courses (*M* = 245 in this study), *xi*, *yi*, and *zi* are translations/rotations at *i* ^th^ time point in the x, y, and z directions, $\Delta t_{x_{i}}=\Delta x_{i}-x_{i-1}$, similar for $\Delta t_{y_{i}}$ and $\Delta t_{z_{i}}$; and ∆*t* represents the FD rotations, ∆d represents the FD translations. Then we also examined group-level head-motion (mean FD) difference between two group using the two-sample t-test. After the above processing, residual time-series were extracted for each voxel and used in subsequent analysis.

### Dynamic Functional Connectivity Strength Analysis

Dynamic FCS of the whole brain voxels were assessed in four frequency bands respectively for each subject. The steps included following: 1) whole time series of one subject was divided into L sliding windows (24, 34), the interval of each window was 5TR (10 s). Since recent studies had shown that the minimum window length of dFC based on sliding window should not be less than 1/_fmin_ (35), thus we selected the time window length of 100 s (_fmin_ =0.01 Hz). We were able to acquire 40 FCS maps for each subject. Next, within the ith time window, the FCS was calculated. 2) In order to exclude the interference of the non-gray matter signals and limit the calculation range, the gray matter template (the GM volume threshold was greater than 0.2) was extracted from the average structure gray matter map of all the subjects. 3) The time course after preprocessing of each voxel was extracted in the gray matter template, and the Pearson correlation coefficient between two voxels time signals was calculated. 4) Fisher's r-to-z transformation was performed. 5) For each voxel in the gray matter template of the brain (brain network node), calculated the sum of the weights (z values) of functional connectivity between this voxel and remaining voxels as nodal FCS. Meanwhile, to avoid the influence of weak correlation functional connections, we set the calculation range to the whole brain voxel with correlation coefficient r > 0.2 (36). 6) In order to better quantify dFCS, we defined the dynamic functional connectivity index of a voxel k as:

$$I_{k}=\frac{\sum_{t=1}^{n}{\left(f_{t}-f_{\mathrm{static}}\right)}^{2}}{n}$$

which came from the previous study (37), where *ft* was the FCS of the k at the time window *t*, *t* = 1,2,…*n*. According to Hui He et al. (37), this dynamic index was an overall metrics, which reflected an average dynamic change relative to its static FCS value. 7) We performed the calculation of this index under the 40 windows each participant and finally obtained the dFCS map. 8) Finally, dFCS maps were apatially smoothed (FWHM = 6 mm).

Firstly, two-sample t-test were used to access group difference in frequency bands respectively. In general, the resting state fMRI signal predominantly distributed in the low-frequency range (0.01–0.1 Hz). Thus, we used repeated measured ANOVA to measure the frequency main effect and interaction effect between frequency and group using SPM12 (www.fil.ion.ucl.ac.uk/spm) in slow-5 and slow-4 frequency bands. During post hoc analysis, we conducted two-sample t-test between groups, and partial t-test between frequency bands. Multiple correction was used for comparison results (false discovery rate correction, p<0.05). During statistical analysis, we controlled age, sex as covariates of no interest. Given previous studies about significant reduction in gray matter (GM) volume of SZ patients (38, 39), we also regressed GM volume during tests.

### Correlation Analysis

In this study, partial correlations between the mean dFCS values of brain regions and the clinical symptoms of the schizophrenia patients were performed. During correlation analysis, to avoid compromising our result, age, gender, and gray matter volume were also considered as covariates to regress.

### Validation Analysis

To keep the robustness of results, validation analysis was performed in randomly selected half of subjects in two groups respectively. Two-sample t-test were used to assess difference between groups in different frequency bands respectively, repeated measured ANOVA were employed to measure interaction effect in slow-5 and slow-4 bands. In addition, we calculated the correlation between drug equivalents of some patients and dFCS of the patients.

## Results

### Demographic Characteristics and Clinical Symptoms

There were no overall significant differences between SZ patients and HCs in distribution of age, gender, mean head motion (two-sample t-test). Of note, only 88 patients had the duration of illness information, and 64 of them participated in the evaluation of PANSS. Detailed participant information was shown in (29).

### The Difference of Dynamic Functional Connectivity Strength Across Frequency Bands in Schizophrenia

**Figure 1:**
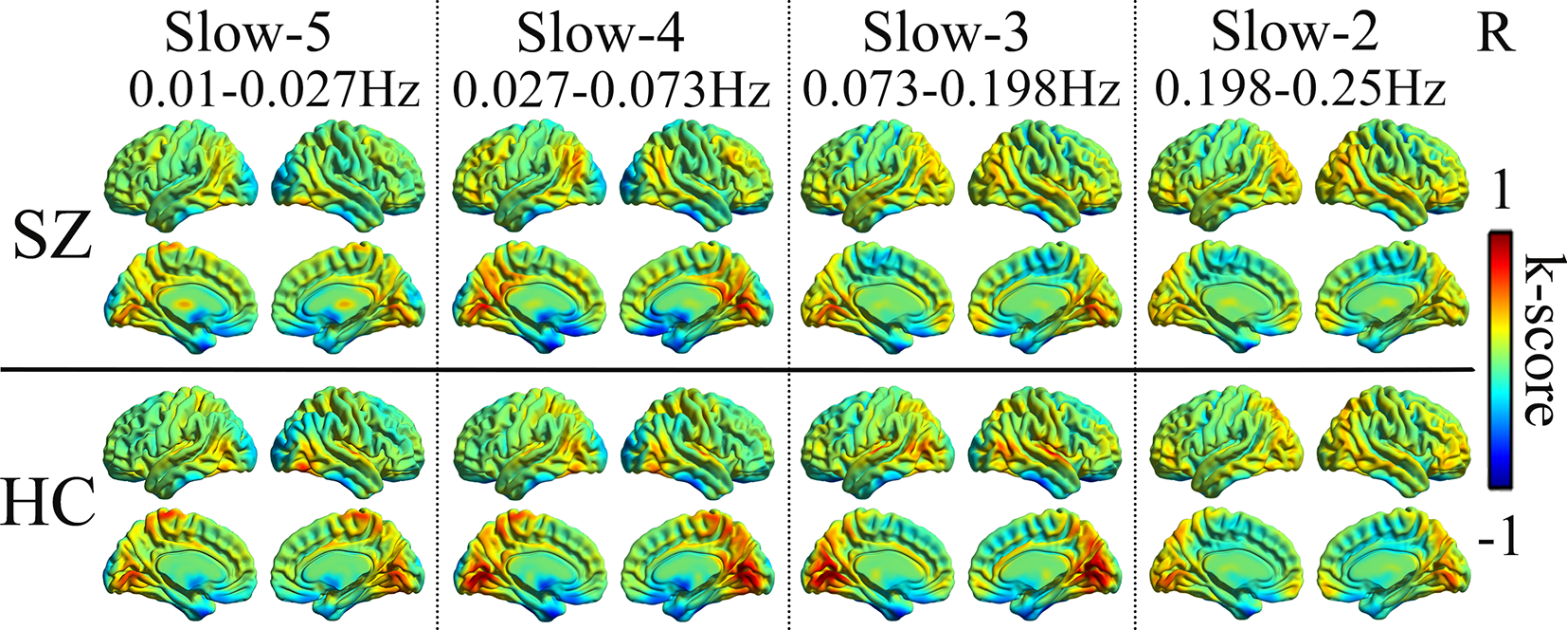
Mean dFCS maps of the SZ and HC in frequency bands from slow-5 to slow-2. dFCS, dynamic functional connectivity strength; SZ, patients with schizophrenia; HC, healthy controls.

In four different frequency bands, the spatial pattern of mean dFCS was similar between SZ and HC groups. As shown in **Figure 1**, the brain network with higher dFCS in slow-5 band mainly included visual network (VN) (lingual gyrus and cuneus), basal ganglia network (BGN) (thalamus and caudate), SMN [postcentral gyrus and supplementary motor area (SMA)], SN [insula and anterior cingulate (ACC)]. Moreover, slow-4 and slow-3 frequency bands were found to be highly similar in schizophrenia patients. They also presented greater dFCS in VN, SN, SMN, the frontal and parietal lobule of frontoparietal network (FPN) (**Figure 1**).

**Figure 2:**
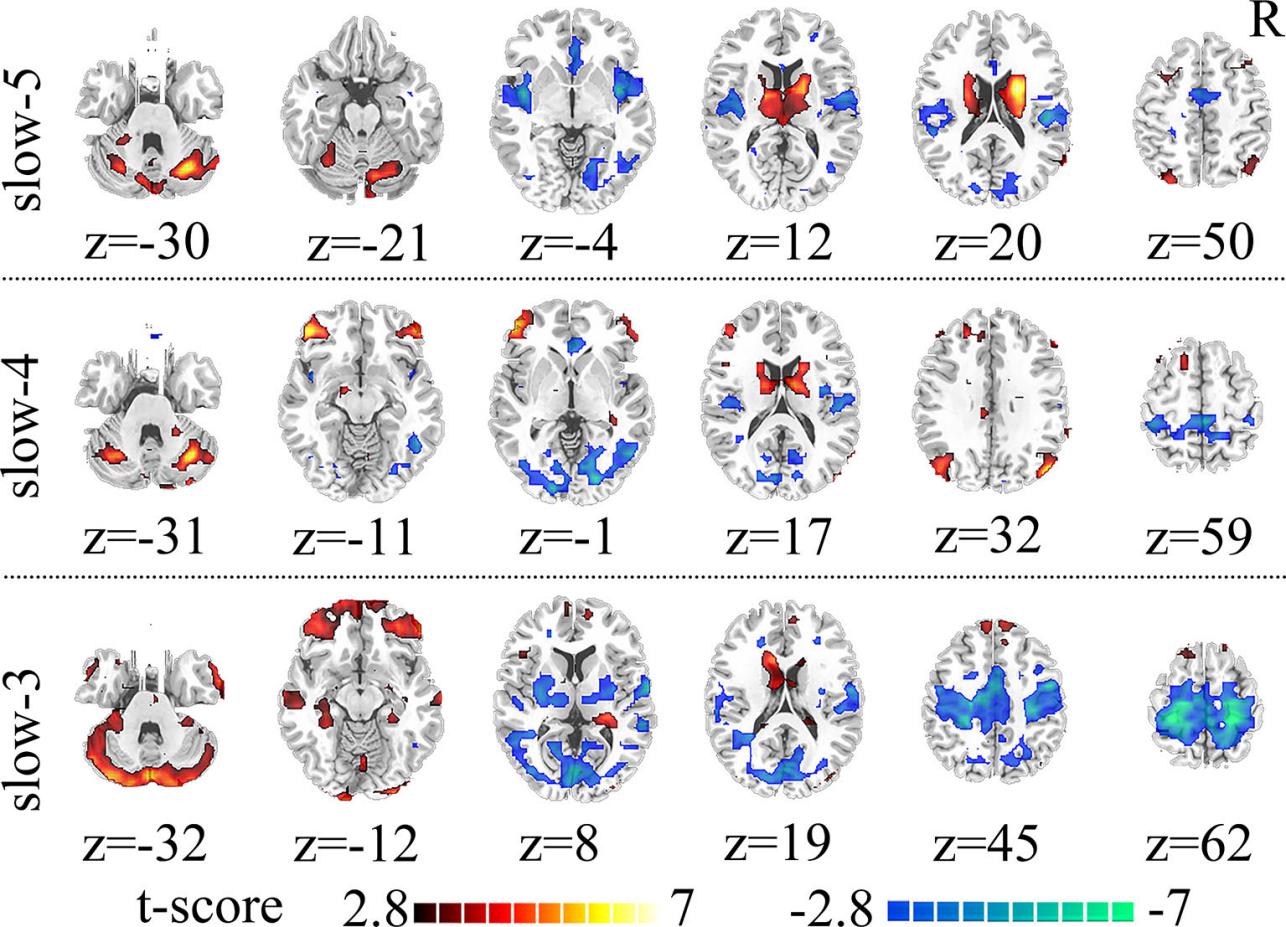
Significant differences in dFCS across frequency bands between the SZ and HC groups. The results were obtained by two-sample t-test. Hot color represents the SZ group had increased dFCS compared with HC group; cold color represents the opposite. Statistical significance level is corrected for multiple comparisons using FDR with (p<0.05, voxels>23). dFCS, dynamic functional connectivity strength; SZ, patients with schizophrenia; HC, healthy controls; FDR, false discovery rate correction.

Compared to HC, SZ patients showed increased dFCS in cerebellum, BGN (thalamus and caudate), DMN (angular gyrus), while decreased dFCS in auditory network (AN) (insula and superior temporal gyrus), SN (anterior insula and ACC), VN (lingual gyrus), SMN (SMA) in slow-5 band. In the slow-4, SZ patients relative to controls exhibited significantly higher dFCS in cerebellum, prefrontal network (PFN), BGN, and DMN. These brain networks major contained: cerebellum crus I and II, orbital middle frontal and orbital inferior frontal gyrus, thalamus and caudate, and bilateral angular gyrus. Nevertheless, in slow-4 frequency band, SZ patients presented lower dFCS in VN (lingual gyrus, cuneus, inferior occipital gyrus), SMN (postcentral gyrus and paracentral lobule), and SN (insula and right ACC). In slow-3 band, significantly increased dFCS were observed in SZ, within cerebellum crus, PFN (medial superior frontal gyrus, orbitofrontal cortex), BGN (middle temporal gyrus and left caudate), hippocampus, and fusiform gyrus. In addition, we also observed decreased dFCS in AN (rolandic operculum and superior temporal gyrus), VN (cuneus, calcarine, and middle temporal gyrus), SMN (precentral gyrus, SMA, and postcentral gyrus). Especially, we found decreased dFCS in thalamus and putamen of BGN in slow-3 (**Figure 2**, **Table 1**). However, there was no significant difference of schizophrenic subjects in slow-2 compared to healthy controls.

**Table 1:** Brain regions with significant differences in dynamic functional connectivity strength (dFCS) across frequency bands (slow-5 to slow-2) based on the two-sample t-test of the results between the patients with schizophrenia (SZ) and health control (HC) groups.

Brain regions (AAL)	Cluster size (voxels)^a^	Peak coordinates (MNI)			T value
		x	y	z	
Slow-5: SZ>HC					
Cerebelum_Crus1_R	1,206	30	−69	−30	7.160
Cerebelum_Crus1_L					
Cerebelum_Crus2_L/R					
Caudate_R	447	17	2	18	6.547
Thalamus_L/R					
Caudate_L					
Angular_R	138	63	−60	21	4.620
Slow-5: SZ<HC					
Insula_R	458	37	0	−5	−6.104
Temporal_Sup_R					
Temporal_Sup_L	397	−42	0	−9	−6.320
Insula_L					
Lingual_R	296	15	−69	−3	−5.070
Cuneus_L/R					
Supp_Motor_Area_R	159	4	−2	50	−4.333
Supp_Motor_Area_L					
Cingulum_Ant_R	73	3	33	−3	−4.770
Cingulum_Ant_L					
Slow-4: SZ>HC					
Cerebelum_Crus1_R	272	30	−72	−27	5.920
Cerebelum_Crus1_L	144	−32	−64	−30	4.506
Frontal_Inf_Orb_L	367	−42	42	−15	5.580
Frontal_Inf_Tri_L					
Frontal_Mid_Orb_L					
Frontal_Inf_Orb_R	171	51	42	−9	5.090
Frontal_Mid_Orb_R					
Caudate_R	244	19	−1	19	4.262
Caudate_L					
Thalamus_L/R					
Angular_R	190	51	−78	27	5.560
Angular_L					
Slow-4: SZ<HC					
Rolandic_Oper_R	217	58	−14	20	−4.380
Insula_R					
Calcarine_R	873	18	−86	6	−5.530
Calcarine_L					
Lingual_L/R					
Occipital_Inf_L/R					
Cuneus_L/R					
Rolandic_Oper_L	101	−42	3	−12	−4.440
Insula_L					
Cingulum_Ant_R	23	3	27	0	−4.490
Postcentral_R	596	9	−48	63	−4.930
Postcentral_L					
Paracentral_Lobule_L/R					
Slow-3: SZ>HC					
Cerebelum_Crus1_L	3,672	−41	−78	−28	5.260
Cerebelum_Crus1_R					
Temporal_Mid_L/R					
Cerebelum_Crus2_L/R					
Temporal_Inf_L/R					
Frontal_Inf_Orb_L/R					
Frontal_Mid_Orb_L/R					
Hippocampus_L/R					
Fusiform_L/R					
Caudate_L					
Frontal_Sup_Medial_L	299	15	18	69	4.950
Frontal_Sup_Medial_R					
Slow-3: SZ<HC					
Postrcentral_R	4,834	24	−33	60	−7.830
Postrcentral_L					
Temporal_Mid_L/R					
Precentral_L/R					
Calcarine_L/R					
Cuneus_L/R					
Supp_Motor_Area_L/R					
Temporal_Sup_L/R					
Rolandic_Oper_R					
Thalamus_R					
Putamen_R					
Putamen_L	204	−30	−12	3	−5.140
Thalamus_L					

MNI, Montreal Neurological Institute; L, left; R, right.

^a^The cluster size represents the number of voxels within the cluster.

### Main Effect of Frequency Factor in Slow-5 and Slow-4

**Figure 3:**
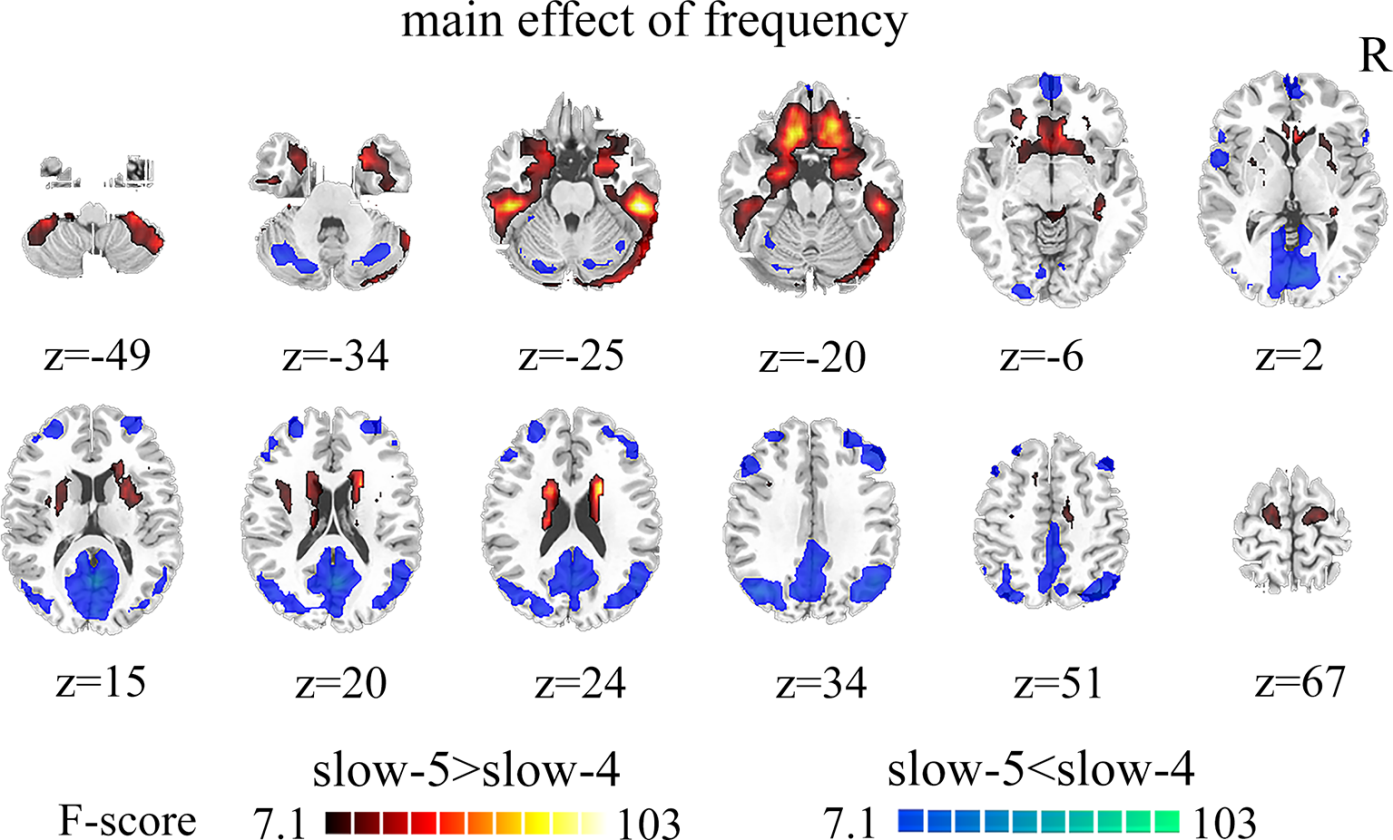
The main effect of frequency band (slow-5, slow-4) across groups on dFCS. The results were obtained by two-way ANOVA (FDR corrected, p < 0.05, voxels > 23). Hot color represents the slow-5 had increased dFCS compared with slow-4; cold color represents the opposite. dFCS, dynamic functional connectivity strength; ANOVA, analysis of variance; FDR, false discovery rate correction.

Brain regions showed a significant main effect of frequency bands, and post hoc analysis indicated that dFCS of slow-5 band were greater than slow-4 band mainly in cerebellum VIII region, BGN, SMN, PFN, SN. The region of BGN includes hippocampus, parahippocampal gyrus, putamen, caudate, superior temporal pole. The SMN primarily contained left paracentral lobule, right precentral, SMA. FPN showed spatial patterns comprising the orbital superior frontal and orbital inferior frontal gyrus, superior frontal. The SN mainly encompassed ACC. However, the brain regions of slow-4 were higher than slow-5 band mainly distributed in VN, FPN, DMN, and cerebellum, which include calcarine, middle occipital and superior occipital gyrus, cuneus, lingual gyrus, left inferior parietal, precuneus, frontal cortex, angular gyrus, cerebellum crus I and II (**Figure 3**, **Table 2**). The main effect of group identified brain regions was similar to those reported in previous analysis using two-sample *t*-test.

**Table 2:** Brain regions with significant main effect of frequency band (slow-5, slow-4) differences in dynamic functional connectivity strength (dFCS).

Brain regions	Cluster size (voxels)^a^	Peak coordinates (MNI)			F-value
(AAL)		x	y	z	
Slow-5<Slow-4					
Calcarine_R	3,869	3	−66	15	53.080
Calcarine_L					
Precuneus_L/R					
Angular_L/R					
Occipital_Mid_L/R					
Cuneus_L/R					
Lingual_L/R					
Cerebelum_Crus1_L/R					
Occipital_Sup_L/R					
Cerebelum_Crus2_L/R					
Parietal_Inf_L					
Frontal_Mid_R	503	42	24	42	25.040
Frontal_Sup_R					
Frontal_Inf_Tri_R					
Frontal_Mid_L	353	−45	24	39	26.180
Frontal_Inf_Tri_L					
Slow-5>Slow-4					
Temporal_Inf_R	2,448	51	−30	−27	103.670
Temporal_Inf_L					
Putamen_L/R					
Cerebelum_8_L/R					
ParaHippocampal_L/R					
Frontal_Sup_Orb_L/R					
Hippocampus_L/R					
Frontal_Inf_Orb_L/R					
Caudate_L/R					
Temporal_Pole_Sup_L/R					
Cingulum_Ant_L					
Supp_Motor_Area_L	173	−15	−12	63	29.600
Paracentral_Lobule_L					
Frontal_Sup_L					
Frontal_Sup_R	127	18	−8	67	18.460
Supp_Motor_Area_R					
Precentral_R					

MNI, Montreal Neurological Institute; L, left; R, right.

^a^The cluster size represents the number of voxels within the cluster.

### Frequency and Group Interaction Effects

**Figure 4:**
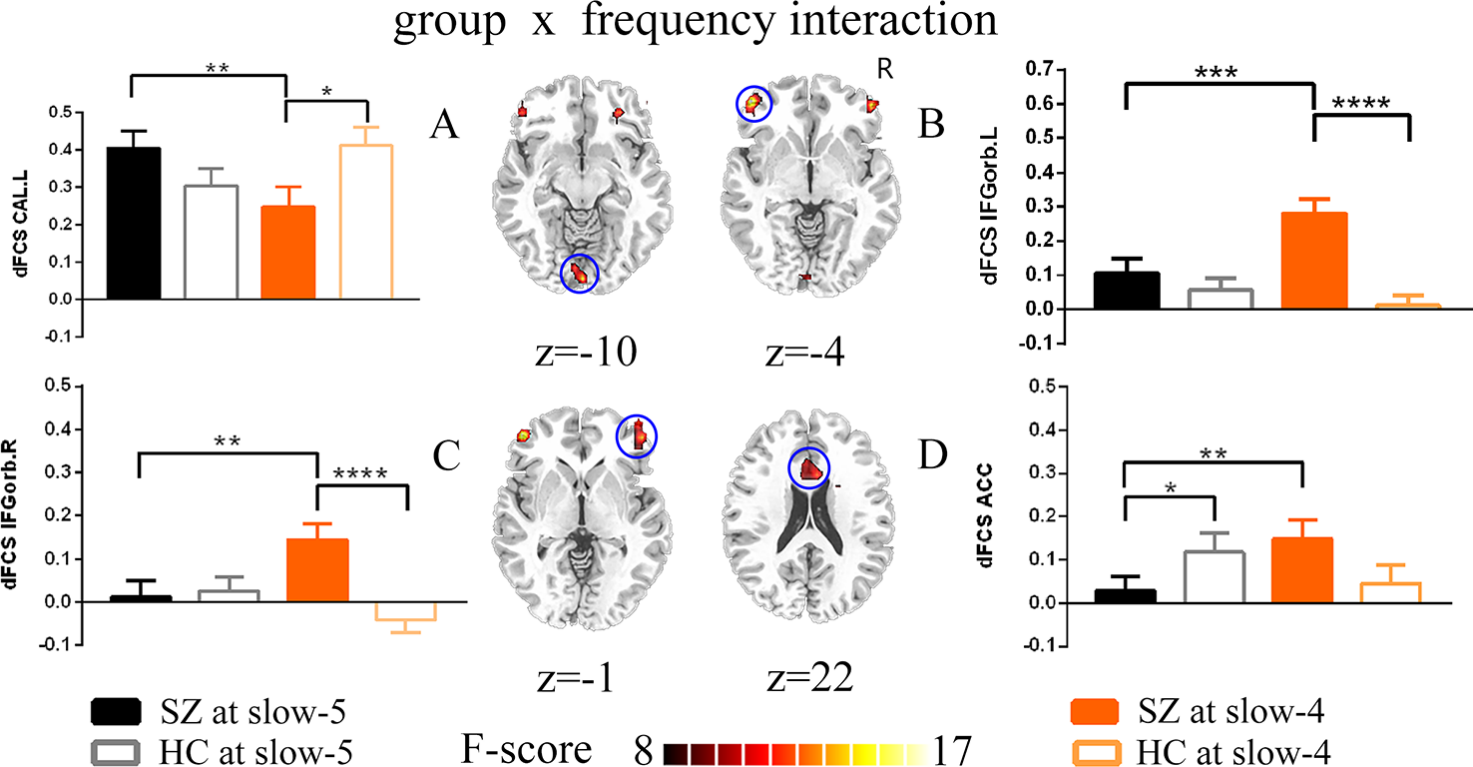
The interaction between the group and frequency band (slow-5 and slow-4) on dFCS. The results were obtained by two-way ANOVA and post-hoc test (p<0.005, uncorrected). **(A–D)** separately represent significant group *frequency interactions and post-hoc test of the left calcarine, the left inferior orbitofrontal cortex, the right inferior orbitofrontal cortex and the anterior cingulum. The data were expressed as the mean value and standard error. *p<0.1, **p<0.05, ***p<0.005, ****p<0.0001. ANOVA, analysis of variance; dFCS, dynamic functional connectivity strength; SZ, patients with schizophrenia; HC, healthy controls; CAL.L, the left calcarine; IFGorb.L, the left inferior orbitofrontal cortex; IFGorb.R, the right inferior orbitofrontal cortex; ACC, the anterior cingulum.

Remarkable interactions between group (SZ and HC) and frequency (slow-5 and slow-4) were found in left calcarine, the bilateral inferior orbitofrontal cortex, and the anterior cingulum (p < 0.005, uncorrected; **Figure 4**, **Table 3**). Further post-hoc tests indicated that within SZ group, the dFCS in left calcarine of slow-4 was significantly lower than slow-5, and in slow-4, the dFCS of SZ group was marginally significantly reduced compared with HCs (p = 0.066, t = −1.849) (**Figure 4A**). Moreover, in the bilateral inferior orbitofrontal cortex, post-hoc two-sample t-test both showed that compared with slow-5, significantly increased dFCS were observed in slow-4 of the SZ group, and the dFCS of patient group was significantly higher than the HC group at slow-4 band (**Figures 4B, C**). In addition, within SZ group, we also found that significant increased dFCS of the anterior cingulum in slow-4. Also, in the slow-5 frequency band, the dFCS of SZ was marginally significantly lower than HC group (p = 0.093, t = −1.687) (**Figure 4D**).

**Table 3:** Significant interaction effects between the frequency band (slow-4 and slow-5) and group by a two-way ANOVA and a post-hoc test.

Brain regions(AAL)	Cluster size (voxels)	Peak coordinates (MNI)			F value
		x	y	z	
Calcarine_L	28	0	−91	−10	15.330
Frontal_Inf_Orb_L	31	−49	46	−4	14.200
Frontal_Inf_Orb_R	40	46	48	−1	16.180
Cingulum_Ant	31	3	29	20	14.440

MNI, Montreal Neurological Institute; L, left; R, right.

### Correlation Between the Dynamic Functional Connectivity Strength With Clinical Symptoms

**Figure 5:**
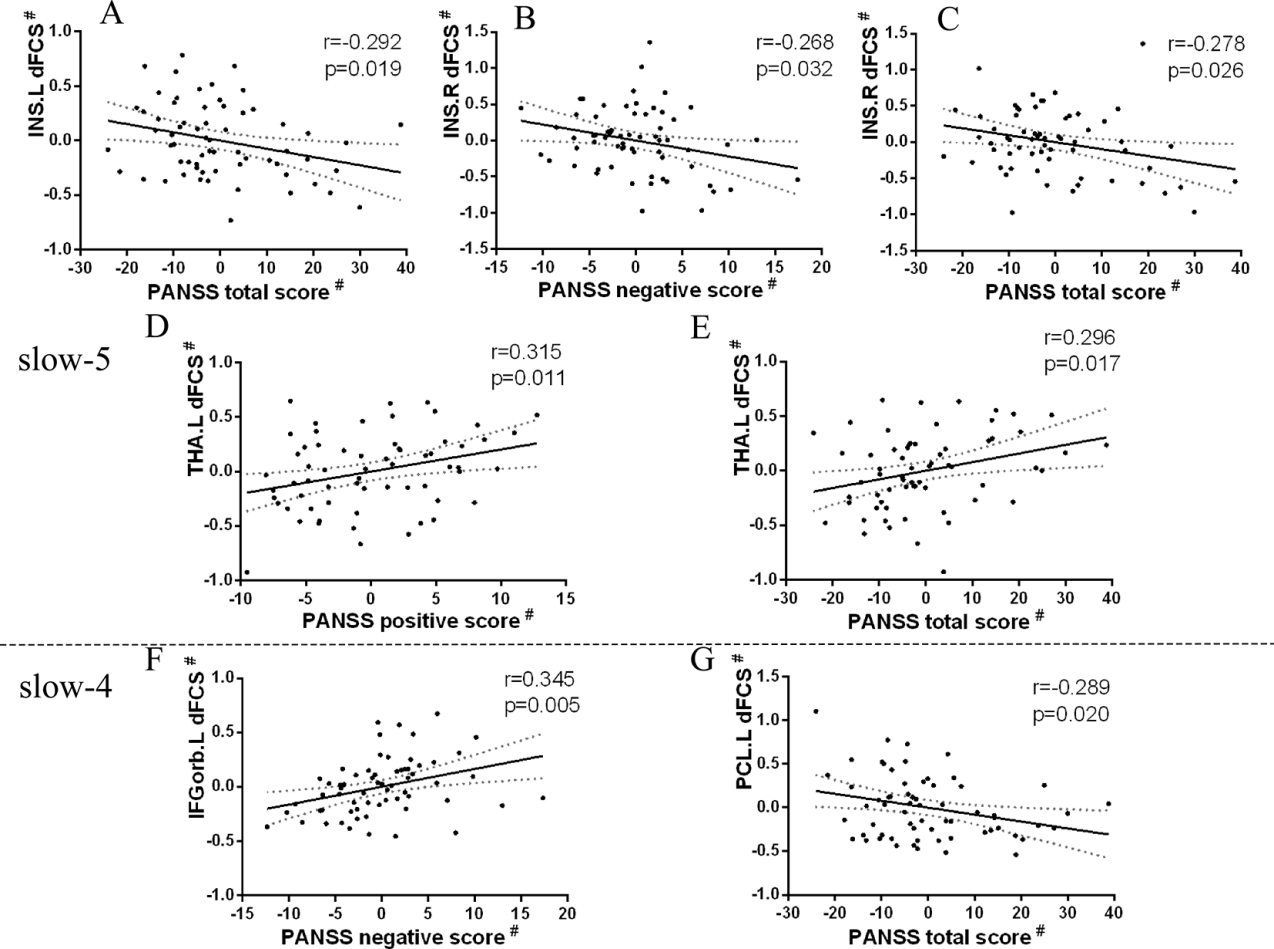
Relationship between regions showing significant two-sample t-test results and clinical scale of symptoms in patients with SZ (p < 0.05). Partial correlations were calculated over the data after regressing covariate (age, sex, GM). # represents the residuals after regression of age, sex, and GM volumes using a general linear model. **(A)** Significant negative correlation between the left insula and PANSS total score in slow-5 band. **(B)** Significant negative correlation between the right insula and PANSS negative score in slow-5 band. **(C)** Significant negative correlation between the right insula and PANSS total score in slow-5 band. **(D)** Significant positive correlation between the left thalamus and PANSS positive score in slow-5 band. **(E)** Significant positive correlation between the left thalamus and PANSS total score in slow-5 band. **(F)** Significant positive correlation between the left inferior orbitofrontal cortex and PANSS negative score in slow-4 band. **(G)** Significant negative correlation between the left paracentral lobule and PANSS total score in slow-4 band. SZ, patients with schizophrenia; GM, the volume of grey matter; dFCS, dynamic functional connectivity strength; PANSS, the positive and negative symptom scale; INS.L, the left insula; INS.R, the right insula; THA.L, the left thalamus; IFGorb.L, the left inferior orbitofrontal cortex; PCL.L, the left paracentral lobule.

**Figure 6:**
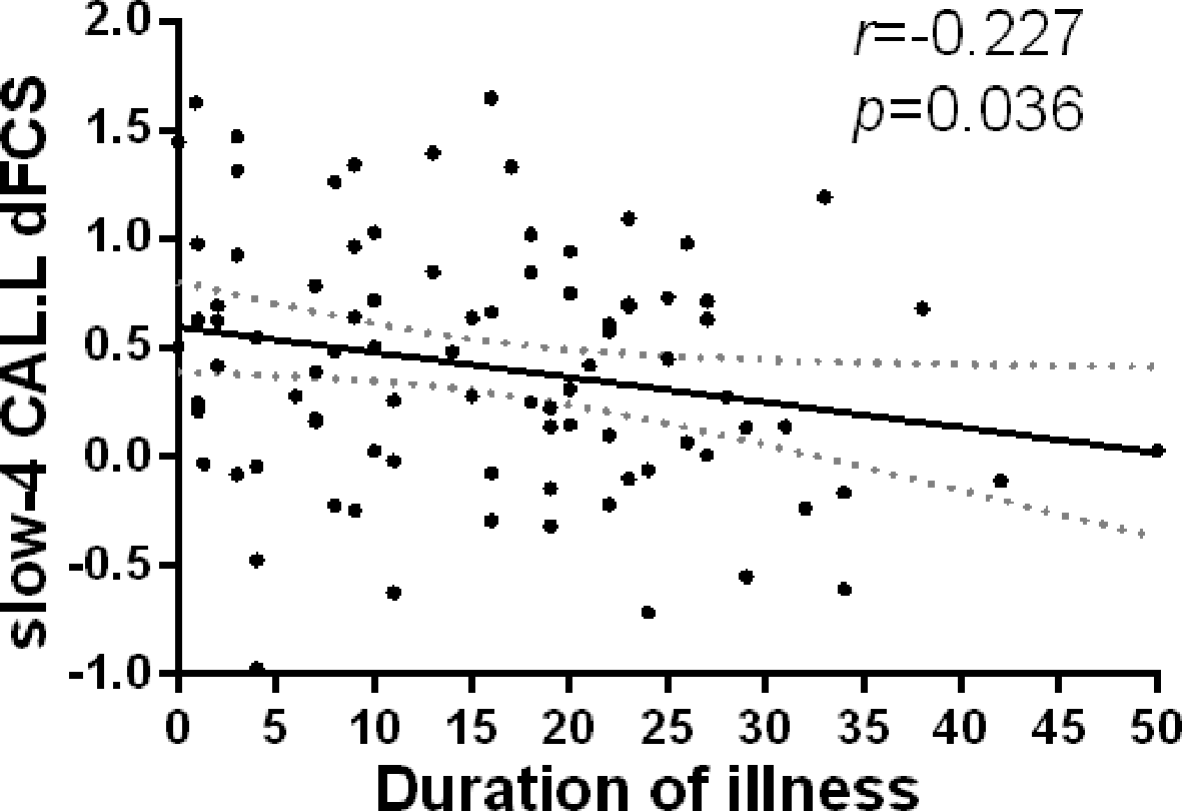
Relationship between regions showing significant interaction effect results and duration of illness in patients with SZ (p<0.05). Partial correlations were calculated over the data after regressing covariate (age, sex, GM). SZ, patients with schizophrenia; GM, the volume of grey matter; dFCS, dynamic functional connectivity strength; CAL.L, the left calcarine.

Firstly, we extracted dFCS values of patients group from significantly interaction effects, their correlation analysis with clinical symptoms showed decreased dFCS of left calcarine was negatively correlated with duration of illness in slow-4 band (p = 0.036, r = −0.227) (**Figure 6**). Then in four different frequency bands, correlation between brain regions of significant group differences and clinical symptoms revealed that decreased dFCS of left insula was negatively correlated with the PANSS total score (p = 0.022, r = −0.292) in slow-5 band (**Figure 5A**). And the dFCS of right insula was negatively correlated with the PANSS negative score (p = 0.037, r = −0.268) (**Figure 5B**) and total score (p = 0.03, r = −0.278) (**Figure 5C**). Moreover, positive correlations between the increased dFCS of left thalamus and PANSS positive score (p = 0.014, r = 0.315) (**Figure 5D**) and total score (p = 0.02, r = 0.296) (**Figure 5E**) were observed in slow-5. Secondly, in slow-4 frequency band of SZ group, a positive correlation between the dFCS of the left orbital inferior frontal gyrus and PANSS negative score (p = 0.006, r = 0.345) (**Figure 5F**) was discovered, and the decreased dFCS of the left paracentral lobule was negatively correlated with PANSS total score (p = 0.024, r = −0.289) (**Figure 5G**).

We found similar results through validation analysis.

## Discussion

Through dFCS analysis, the current study investigated the difference of frequency-dependent whole brain dynamic functional connection in schizophrenia. Consistent with our hypothesis, significantly altered dFCS of SZ mainly distributed in cerebellum, VN, AN, SN, PFN, SMN, DMN, and BGN in different frequency bands. Interesting, compared to HC group, the thalamic subregion of SZ group exhibited enhanced dynamic FCS in slow-5 and slow-4, while reduced in slow-3. Moreover, in slow-5 and slow-4, significant frequency-by-group interaction effect were observed in the left calcarine, the bilateral inferior orbitofrontal gyrus and ACC. Furthermore, the altered dFCS of insula, thalamus, calcarine cortex, orbitofrontal gyrus, and paracentral lobule were partial correlated with clinical symptoms of SZ patients in slow-5 and slow-4 bands respectively. Together, these results demonstrate that altered dFCS in the SZ patients distribute throughout the whole brain, and the abnormal dynamic changes is frequency dependent. These findings may offer more evidence to explore the underlying pathological mechanism of SZ.

### Altered Dynamic Functional Connectivity Strength Across Four Frequency Bands in Schizophrenia

Previous studies suggested that decreased functional connectivity (FC) of somatosensory and visual network were a potential mechanism for self-disorder in SZ (40). Kraepelin and Bleuler firstly found sensorimotor processing dysfunction and integration defect of multiple sensory functions in patients with SZ, and they put these as a possible psychopathological mechanism in SZ (41). Moreover, the lower FC of visual network in multiple frequency bands had been reported in several researches (13, 42, 43). These researches indicate disturbed abnormally integration in perception-motor processing of SZ. In the current study, the reduced dFCS of the somatosensory and visual network in slow-5, slow-4, and slow-3 may make it difficult for patients to perceive their own state. The significantly negative correlation between reduced dFCS of insula, paracentral lobule, and clinical symptoms suggest that abnormal processing of perceptional information in SZ helps us to understand the pathological mechanisms involved self-disorder. Moreover, we also found increased dFCS of cerebellum and BGN in different frequency bands. Cerebellum and BGN were considered as a partially functional and strongly connected nervous system (44), a functional abnormality in one network will have the same impact on another neural network (45, 46). Therefore, abnormality in cerebellar function in patients with SZ may be strongly correlated with abnormality in the BGN. The current findings reveal that abnormal cerebellar and BGN functional connections may play a crucial role in cerebellar-BGN-cortical network circuits and may lead to dysfunctional interaction between the subcortical network and the cerebral cortex network in patients with SZ. Especially, the abnormal functional integration of this loop is modulated by frequency band.

Interestingly, we found the dFCS of subregion thalamus presented an imbalance in different frequency bands of SZ subjects. The thalamus is a crucial node for brain physiology implicating in processing of sensorial inputs and emotion processing (47, 48). The BGN received incoming signals from different regions of the brain and projected them back through the thalamus to the cortex. Recently, altered thalamus and cortico-thalamic dysconnection had been revealed in patients with SZ (49–51). Our study extended previous findings to higher frequency band, interestingly, the different thalamic subregion of schizophrenic patients exhibited enhanced dynamic FCS in slow-5 and slow-4, while reduced in slow-3. Besides, we also found that the dFCS of the left THA was positively correlated with PANSS positive/total score in slow-5 of SZ subjects. This imbalance suggests that the dynamic abnormality of THA in patients with SZ may be modulated by frequency band, and thalamus showed competitive signals in different frequency bands, which may be related abnormal affective perception and higher cognitive processing in patients with SZ. In the future, the function of thalamic subregion in different frequency bands should be explore.

### Frequency Specific Changes in Dynamic Functional Connectivity Strength Schizophrenia (Slow-5, Slow-4)

In the main effect of the frequency band (slow-5 and slow-4), we found that the cerebellum, BGN, SMN, PFN, and SN had higher dFCS in slow-5, while VN, FPN, DMN had stronger dFCS in slow-5. The cerebellum plays an essential role in the functional interaction between the cortex and the subcortex. The dorsolateral prefrontal lobe (DLPFC) and posterior parietal lobes are the core nodes of the frontoparietal network (FPN). Some neuroimaging studies found that cerebellum and FPN are involved in advanced cognitive information processing (45, 52). Our results indicate that the process of high-frequency information processing in the cerebellum and FPN may enhance the efficiency of advanced cognitive processing. During the processing of cognitive information, the cerebellum, as an unsupervised regulatory brain region, no need the feedback of the higher network. Conversely, the striatum system, particularly the caudate nucleus and frontal cortex, conduct some supervised neuro modulation of speech, memory, mood, and behavior (53–55). Moreover, higher low frequency signals may improve the stability of supervisory regulation during the processing of information and high frequency signals. Therefore, in future researches of schizophrenia, we should consider the frequency band information.

In the visual cortex, orbitofrontal cortex (OFC) and ACC, frequency-by-group interactions between SZ patients and HC controls were observed in our study. The PFN was considered crucial for higher cognitive functions of the brain, such as working memory, control of goal-directed, integration, and coordination of complex cognitive activities (56), OFC and ACC were considered the part of PFN (57). The OFC is known to be closely related to emotional expression, decision making, hedonic experience, and plays a key role in tasks requiring response inhibition (58–60). The ACC is the part of medial PFN, and is critical for executive attention, regulation of motivational and emotional behavior (61). Abnormalities of the OFC and ACC in schizophrenia patients have been reported in several studies (62–64), and these impairments appear to be with the core disease pathophysiology (65). Consistent with previous studies, we also found abnormalities of OFC and ACC in SZ in current study, interestingly, in our study, these dynamic changes were modulated by frequency band. Within SZ group, the dFCS in OFC and ACC of slow-4 was both significantly greater than slow-5 band. The two brain regions of patients have abnormal dynamic characteristics of reduced low frequency signal and increased high frequency signal in their respective frequency bands, which may affect the failure of executive attention, regulation of motivational and emotional behavior, further affect organization and control of goal-directed thought in PFN.

Moreover, compared with HC controls, in slow-5 of the current study, the dFCS of schizophrenic subjects ACC was reduced, while increased dFCS of the OFC in slow-4. A recent study suggested that in brain functional networks, physiological signals in different frequency bands could compete or interacted with each other in the same functional network (66). For the cognitive function of PFN, researchers suggested that lateral PFC was responsible for selection, monitoring and manipulation, medial PFC updated tasks, and OFC conferred social and emotional significance to tasks (61). Thus, our results suggest OFC and ACC interconnect in different frequency bands, their dysfunction may lead to the regulation disorder of cognition and emotions in SZ patients, further some abnormal behavioral.

Previous studies suggested that reduced activation region in the visual magnocellular pathway was related to the deficits in motion processing in schizophrenia (67). Converging evidence suggests that the high frequency visual information collection and transmission may help the bottom-up information integration of the brain. In this study, we observed interaction effect in visual network and the significant negative relationships between dFCS in the left calcarine and duration disease (**Figure 6**). We also found that the dFCS of SZ visual cortex was reduced in slow-4, while the opposite in HC group. Namely, our finding suggest that the reduction of high-frequency dynamic information in primary visual cortex of SZ may affect the collection and transmission of primary information, and may further affect its advanced cognitive function.

## Limitations

Some limitations should be taken into account in the current study. Firstly, of note, most of patients with schizophrenia were taking the medication (e.g., antipsychotics). Although we assessed the correlation between drug equivalents of some patients and dFCS of these patients, the results showed no significant correlation, we could not rule out whether the antipsychotic medications has an effect on the observed findings. Thus, in future study, we should validate our findings in first-episode schizophrenic patients. Secondly, although we had regressed 12 head motion parameters, it was still possible that some of the results were affected by motion-induced artifact. Finally, BOLD fMRI signal in high frequency (>0.1 Hz) usually considered physiological noises (respiration and cardiac signal) and are regressed, however, recent studies had confirmed that high-frequency information in resting-state fMRI signal may provide useful information in clinical studies (68, 69). In our study, we were lacked of collecting the physiological signals (respiration and cardiac signal) of subjects, so we couldn't use the general linear model (GLM) to remove the physiological signal noise, there might be some influence. In future research, we will collect physiological signal and conduct further analysis.

## Conclusions

In this study, we found the altered dFCS within the brain of SZ patients was sensitive to specificity of frequency band. Significantly abnormal dFCS of schizophrenia mainly within cerebellum, SN, FPN, AN, SMN, VN, BGN, and PFN. Specially, the dFCS of subregion thalamus presented an imbalance in different frequency bands of SZ subjects, and the dFCS of calcarine cortex, insula, THA, OFC, PCL were associated with clinical symptoms, implying that the symptoms were related to abnormally dFCS at specific frequency bands. Moreover, in slow-5 and slow-4, significant frequency-by-group interaction effects were observed in the left calcarine, the bilateral inferior orbitofrontal gyrus and ACC. Our results may provide potential implications for exploring the neuropathological mechanism of schizophrenia.

## Data Availability Statement

The datasets generated for this study are available on request to the corresponding author.

## Ethics Statement

The studies involving human participants were reviewed and approved by the Ethics Committee of the Clinical Hospital of Chengdu Brain Science Institute. The patients/participants provided their written informed consent to participate in this study.

## Author Contributions

DY, JL, CL, and HHe designed and checked the study. HHu, ZH, and YL analyzed the imaging data. YL wrote the first manuscript. YL, HHe, MD, HW, GY, DY, JL, and CL participated in the conception, drafting, and revision of the article. All authors contributed to and gave final approval of the manuscript.

## Funding

This work was partly supported by a grant from the National Key R&D Program of China (2018YFA0701400) and grants from the National Natural Science Foundation of China (grant numbers: 61933003, 61761166001, 81471638, 81571759).

## Conflict of Interest

The authors declare that the research was conducted in the absence of any commercial or financial relationships that could be construed as a potential conflict of interest.
